# The Long-Term Care Insurance System in Japan: Past, Present, and Future

**DOI:** 10.31662/jmaj.2018-0015

**Published:** 2019-02-01

**Authors:** Masao Iwagami, Nanako Tamiya

**Affiliations:** 1 Department of Health Services Research, Faculty of Medicine, University of Tsukuba, Tsukuba, Japan

**Keywords:** long-term care, community-based integrated care system, health services research

## Abstract

The Japanese population is rapidly aging. The proportion of people aged ≥65 was 27.3% in 2016, the highest in the world. Japan achieved universal health coverage for medical care in 1961 with the introduction of the National Health Insurance (NHI) system. However, increasing expenditure on inpatient care for old people became a significant issue in society. At that time, tax-supported in-home services were mainly for old people with low incomes and little care given by family. To tackle these problems, universal health coverage for long-term care was introduced in 2000 under the Long-Term Care Insurance (LTCI) system. People aged ≥65 who satisfied the eligibility criteria and those aged 40–64 with age-related diseases are entitled to receive long-term care services at home or in facilities, irrespective of income level and availability of family caregiving. The practical benefits in kind under the LTCI system for family caregivers have been demonstrated. However, because of a recent increase in long-term care costs, especially facility-based costs, it may be necessary to give more support to family (informal) caregivers who participate in home-based long-term care. Health services research using nationwide claims data would help sustain the LTCI system through evidence-based policymaking. Recent studies have explored how to prevent deterioration of care need levels among residents of long-term care welfare facilities and how to promote a shift from facility-based to home-based long-term care services. By 2025, as the baby boomer generation is projected to reach the age of 75, the Japanese government is planning to establish a community-based integrated care system. Harmonization between long-term care and medical care, involving the informal sector and nonprofit organizations, would mitigate the increasing cost of both the NHI and LTCI systems. To achieve this, more research is warranted to understand how long-term care, medical care, and informal care can be effectively integrated in the community.

## Japan’s Aging Population

The Japanese population has been rapidly aging in the past decades because of decreasing birth rates and increasing life expectancy. According to the latest statistics in 2016, the life expectancy of Japanese men and women was estimated to be 81.0 and 87.1 years, respectively ^(1)^. The proportion of people aged ≥65 was 27.3% in 2016, the highest in the world. The doubling time of aging, defined as the time it takes for the proportion of people aged ≥65 to increase from 7.0% to 14.0%, was 24 years (from 1970 to 1994) in Japan, compared to 40 years in Germany and 115 years in France ^(2)^.

## The National Health Insurance and Long-Term Care Insurance Systems in Japan

In 1961, Japan achieved universal health coverage for medical care with the introduction of the National Health Insurance (NHI) system ^(3)^. In 1973, the system started to provide free medical care (i.e., no co-payment) for everyone aged ≥70. Although free medical care was ended in 1983, the co-payment rate was set low for old people. Meanwhile, there was an increasing trend in the number of nuclear families due to urbanization ^(4)^. Consequently, many old people were admitted and remained in hospitals because their families were unable or unwilling to care for them ^(5)^. This was called “social admission,” and the associated increase in the medical expenditure on inpatient care became a significant issue in society. At the time, tax-supported in-home services were mainly for old people with low incomes and little care given by family. Although the Japanese government implemented a ten-year strategy for the health and welfare of the elderly (the “Gold Plan”) in 1989, the system encountered a financial issue due to its reliance on tax revenue and its increasing expenditure.

To tackle these problems, universal health coverage for long-term care was introduced in 2000 under the Long-Term Care Insurance (LTCI) system ^(5), (6)^. The aims of establishing the LTCI system included shifting the burden of family caregiving to social solidarity, shifting cost sharing via an insurance premium system, and integrating long-term medical care and welfare services. Under the LTCI system, people aged ≥65 who satisfied the eligibility criteria, as well as those aged 40–64 with age-related diseases, can receive long-term care services, irrespective of income level and availability of informal care provided by the family ^(5), (6)^. Eligibility is assessed using a 74-item questionnaire based on activities of daily living, and there are seven levels of long-term care need certificates: support levels 1 and 2 and care need levels 1 (least disabled) to 5 (most disabled). The availability of family caregiving is not taken into consideration, even though it was the key assessment point in the previous system. Insurance benefits in kind include in-home services (e.g., home visits/day services and short-stay services/care) and services at facilities, including long-term care welfare facilities (also called special nursing homes, or *tokubetsuyougoroujinhoumu* or “*tokuyo*” in Japanese), long-term care health facilities (also called geriatric health services facilities, or *roujinhokenshisetsu *or**“*roken*” in Japanese), and long-term care medical facilities (medical long-term care sanatoriums, or *kaigoryouyougatairyoushisetsu* or “*ryoyogata*” in Japanese), and do not include cash benefits or other direct benefits for family caregivers (e.g., pension and worker’s compensation) to encourage the use of the services in kind.

## Effects of the Introduction of the LTCI System on Family Caregivers

Tamiya et al. demonstrated a sharp increase in formal service use by the frail aged population, especially people in the high-income group, after the introduction of the LTCI system ^(5)^. The study also showed that the average time spent on caring by family caregivers decreased significantly after the introduction of LTCI, whereas the time spent on working increased significantly only in the high-income group. It might be difficult for family caregivers in the low- and middle-income groups to find a job that can be continued while caring even under the support of LTCI.

After over 18 years from the introduction of the LTCI system, direct benefits for family caregivers, such as cash, pension, and workers’ compensation, are not available in Japan, although these are provided in Germany and some other countries. The issue of how to respect and support family (informal) caregivers who can participate in home-based long-term care may be becoming increasingly important in this era of rising costs associated with facility-based long-term care services.

## Health Services Research for Better Long-Term Care

It is important to assess the quality and cost-effectiveness of long-term care. For example, using data from a long-term care insurer in a city in Japan, Olivares-Tirado et al. examined the factors associated with long-term care expenditure, the effect of a new preventive policy (implemented in 2005) on long-term care expenditure, and the effect of in-home and community-based services on the functional status of old people ^(7), (8)^. Such studies provide clues to improve the quality of long-term care and to sustain the LTCI system, although the generalizability of their findings in a single city to the whole country might be somewhat limited.

More recently, nationwide LTCI claims data have been made available to selected researchers. Health services research using these data would help sustain the LTCI system through evidence-based policymaking. For example, Jin et al. conducted a study on how to prevent deterioration of care need levels among residents of long-term care welfare facilities ^(9)^. The study revealed that older age and lower care need levels at baseline were associated with deterioration in care need level, whereas metropolitan facilities, unit care model facilities, and mixed care model facilities were less likely to experience care need level deterioration. Furthermore, the study indicated that the proportion of registered nurses and dietitians could be potentially modifiable factors to prevent deterioration of care need level in long-term care facilities. To give another example, Morita et al. conducted a study on how to promote a shift from facility-based to home-based long-term care services ^(10)^. The study revealed that only 19 percent of residents in long-term care health facilities were discharged to their homes during the two-year study period. Older age, a higher level of care need, having several medical conditions, private ownership of the facility, more beds in the facility, and more long-term care facility beds per 1,000 adults aged ≥65 in the region were significantly associated with lower likelihood of being discharged to their home. Therefore, policymakers may need to consider the consolidation of small facilities and appropriate allocation of long-term care beds in each region.

The Japanese government is currently planning to establish a community-based integrated care system that will comprehensively ensure the provision of health care, nursing care, prevention, housing, and livelihood support ^(11)^. With this system, old people are expected to be able to live the rest of their lives in their own way in environments familiar to them, even if they become heavily dependent on long-term care. Moreover, harmonization between long-term care and medical care would mitigate the increasing cost of both in the NHI and LTCI systems. Anecdotally, some people frequently move between long-term care facilities and hospitals, which incur the costs of long-term care and medical care. If these people were managed more appropriately in long-term care facilities, a part of hospitalizations would be prevented. Indeed, using merged medical care and long-term care insurance claims data from a city in Japan, Boyoung et al. estimated that during a year and a half study period, 16.3% and 9.5% of residents in special nursing homes and geriatric health services facilities, respectively, experienced potentially avoidable hospitalizations ^(12)^. Conversely, hospital discharge conference including in-hospital staff and medical and long-term care providers in the community is expected to prevent readmission to the hospital, although the effectiveness of this approach needs to be confirmed by research.

In addition, the informal sector and nonprofit organizations (e.g., senior clubs, residents’ associations, and volunteer groups) are expected to play an important role in the community-based integrated care system. The community networking would provide old people with the potential to maintain their physical and cognitive functions. This would ultimately help to prevent the initiation of long-term care, as well as the excessive use of medical care.

However, only a few studies have focused on this area to date, mainly because linked data between long-term care, medical care, and other health records are unavailable at the national level. As mentioned above, nationwide LTCI claims data are currently available for research, whereas the national database of health checks and health insurance claims (the Japan National Database) is also available for selected researchers. The Japanese government has launched the Data Health Reform Promotion Plan to make nationwide linked electronic health records available, which will be the biggest database on the aged population in the world. Research using these nationwide datasets and evidence-based policymaking would be imperative to achieve community-based integrated care and to sustain the NHI and LTCI systems. Evidence from Japan would also provide important insights for other countries with aging populations.

## Article Information

### Conflicts of Interest

None
